# Beyond single stressors/stimuli in adaptive physiology: Dose–response relationships and translational challenges

**DOI:** 10.1113/EP093875

**Published:** 2026-04-22

**Authors:** Franck Brocherie, Yohan Rousse, Grégoire P. Millet

**Affiliations:** ^1^ Laboratory Sport, Expertise and Performance (EA 7370) French Institute of Sport (INSEP) Paris France; ^2^ University Paris Cité Paris France; ^3^ Institute of Sport Sciences, Faculty of Biology and Medicine University of Lausanne Lausanne Switzerland

**Keywords:** clinical practice, cross‐adaptation, heat stress

To the Editor,

We thank O'Connor et al. (2025a) for their thoughtful letter extending our viewpoint entitled, *‘Turning up the heat: Can thermal therapy really protect muscle health in older adults?’* (Brocherie et al., 2025). Their contribution provides valuable translational evidence demonstrating that passive heating can acutely improve functional exercise capacity in older adults with heart failure with reduced ejection fraction (O'Connor et al., 2025b). The observed improvements in endurance performance, together with increases in femoral artery blood flow and *gastrocnemius* oxygenation, reinforce the physiological rationale we discussed (Brocherie et al., 2025). In particular, elevations in muscle temperature might enhance calcium kinetics, accelerate contractile processes and increase peripheral perfusion, mechanisms that collectively support improved neuromuscular function.

Importantly, these findings move the discussion beyond mechanistic observations and towards clinically meaningful outcomes. But they also highlight a recurring challenge within integrative physiology and clinical/rehabilitation science: translating promising/new physiological/biological insights into practical interventions that remain effective, safe and scalable in real‐world settings.

## 1 STRESSOR/STIMULUS‐BASED ADAPTIVE PHYSIOLOGY

Many interventions designed to enhance functional capacity rely on the controlled application of physiological/biological stressors/stimuli. Passive heat exposure represents one such stressor/stimulus, but it belongs to a broader category that encompasses exercise, hypoxia, cold exposure and dietary restriction. These stressors/stimuli‐based adaptive physiology share a common conceptual basis in hormesis (Calabrese & Baldwin, 2001) or general adaptation syndrome (GAS) (Selye, 1950), which describes how an organism responds to a stressor/stimulus through sequential/successive stages of alarm, resistance (corresponding to a mild/transient stressor/stimulus activating adaptation) and exhaustion (in the event of an excessive stressor/stimulus). Within such a framework, the beneficial performance and/or therapeutic value of a stressor/stimulus depends crucially on its dose (intensity × duration), recovery and frequency, ensuring that exposure remains within an adaptive window rather than being detrimental to the capacity to respond, participating in construction of antifragile physiological/biological systems. In ageing and chronic diseases, conditions frequently characterized by diminished adaptive reserve, carefully dosed stressor/stimulus‐based interventions, such as passive heat exposure, might be viewed not simply as therapeutic treatments targeting isolated physiological impairments but rather as strategies aimed at restoring or reinforcing the capacity of the organism to adapt.

## 2 BEYOND A SINGLE STRESSOR/STIMULUS: DOSE–RESPONSE, COMBINATION AND ADAPTIVE INTERACTIONS

Recognizing passive heat therapy as a hormetic/GAS stimulus naturally raises the question: how should such stressors/stimuli be integrated with other adaptive stressors/stimuli that are already central to clinical/rehabilitation practices? In real‐world settings, individuals are rarely exposed to a single isolated stressor/stimulus; rather, multiple physiological stressors/stimuli interact across overlapping physiological/biological systems.

Exercise training itself represents the typical hormetic/GAS intervention, inducing widespread adaptations across skeletal muscle, cardiovascular and metabolic pathways. In this context, heat exposure might act as a physiological primer, complementing these responses through several mechanisms (accelerated enzymatic activity, increased peripheral perfusion/oxygen delivery and improved neuromuscular function/contractile efficiency), potentially allowing individuals to sustain comparable or even higher workloads at lower perceived effort and/or for longer duration after adequate exposure.

Other environmental stressors/stimuli, such as hypoxia, have also been explored as adjuncts to exercise training, mainly through erythropoiesis, angiogenesis and mitochondrial signalling pathways (Rousse et al., 2026). When considered alongside (passive) heating, such stressors/stimuli raise intriguing/interesting possibilities regarding combined exposure to stressors/stimuli (i.e., including simultaneous exposure, whereby both stressors/stimuli are imposed at the same time; concurrent exposure, whereby they are applied within the same day or phase but in distinct sessions; and sequential exposure, whereby acclimatization to one stressor/stimulus precedes the other over several days) (Rousse et al., 2026), such that complementary (cross‐adaptative) mechanisms can be targeted across multiple levels of the integrative physiological/biological systems.

However, the integration of multiple stressors/stimuli introduces considerable complexity (Rousse et al., 2026). As previously mentioned, physiological/biological adaptations are sensitive to dose–response relationships and duration of exposure, with interactions between stressors/stimuli altering both the magnitude and direction of adaptive responses. If multiple stressors/stimuli are applied simultaneously or at excessive intensity, the cumulative physiological strain might exceed the adaptive capacity of the individual, particularly in older adults or clinical populations, and might induce additional unintended responses (e.g., dehydration resulting from combined exercise and heat exposure). Conversely, with appropriate dose and combination, these stressors/stimuli might reinforce each other's adaptive signals. However, whether such stressors/stimuli should be applied simultaneously, concurrently or sequentially remains largely unresolved and represents an important area for future investigation (Rousse et al., 2026). Adopting a system‐level perspective might therefore be valuable, in particular in ageing and clinical populations, where research might benefit from exploring integrated adaptive strategies that leverage complementary stress pathways while respecting the limits of individual tolerance, rather than viewing each stressor/stimulus in isolation.

## 3 TRANSLATIONAL CHALLENGES IN CLINICAL PRACTICE

Although these concepts are appealing from a physiological/biological perspective, their translation into clinical/rehabilitation practice remains challenging (Morris et al., 2011). Many promising strategies derived from experimental physiology have historically struggled to achieve widespread implementation because practical considerations (e.g., patient heterogeneity, safety concerns and logistical constraints) were insufficiently addressed during early stages of development. O'Connor et al. (2025b) highlighted an important step towards bridging this gap. Demonstrating that passive heating can be implemented safely in individuals with heart failure suggests that environmental stressors/stimuli‐based intervention/therapy might be feasible even in populations with substantial cardiovascular limitations. Nevertheless, broader adoption will require protocols that are effective, safe, scalable and compatible with existing clinical/rehabilitation programmes. Addressing these challenges will therefore require collaborative efforts that combine mechanistic physiology/biology with pragmatic clinical/rehabilitation research and implementation science. Engaging clinicians and patients in the development of thermal protocols might help to ensure that interventions are not only physiologically effective but also acceptable and sustainable in real‐world care settings.

## 4 CONCLUSION

Heat exposure represents a promising example of how controlled stressors/stimuli might be harnessed to enhance adaptive capacity in ageing and clinical/rehabilitation populations. Viewed through the frameworks of hormesis or GAS, heat exposure might contribute to the construction of antifragility within complex physiological/biological systems. Future research should therefore extend beyond examining the isolated effects of (passive) heating and explore how environmental stressors/stimuli interact with other adaptive stressors/stimuli. Determining the optimal dose and combination (e.g., simultaneous, concurrent or sequential) of these stressors/stimuli will be crucial for translating promising physiological/biological concepts into effective and practical clinical and rehabilitation programmes.

## AUTHOR CONTRIBUTIONS

All authors have read and approved the final version of this manuscript and agree to be accountable for all aspects of the work in ensuring that questions related to the accuracy or integrity of any part of the work are appropriately investigated and resolved. All persons designated as authors qualify for authorship, and all those who qualify for authorship are listed.

## CONFLICT OF INTEREST

None declared.

## FUNDING INFORMATION

None.
